# Supplementary material for: Association between Biomarkers of Cardiovascular Diseases and the Blood Concentration of Carotenoids among the General Population without Apparent Illness
Source: Nutrients. 2020 Jul 31;12(8):2310. doi: 10.3390/nu12082310 (PMC7469056; doi:10.3390/nu12082310)
Supplement: Supplementary file 1 [file nutrients-12-02310-s001.zip › nutrients-837869-supplementary.docx]

| **Carotenoid** | | **ρ** |  |  |
| --- | --- | --- | --- | --- |
| Lutein | Zeaxanthin | 0.627 | *** | |
| Lutein | β-Cryptoxanthin | 0.444 | *** | |
| Lutein | α-Carotene | 0.381 | *** | |
| Lutein | β-Carotene | 0.505 | *** | |
| Lutein | Lycopene | 0.031 |  |  |
| Zeaxanthin | β-Cryptoxanthin | 0.303 | *** | |
| Zeaxanthin | α-Carotene | 0.182 | *** | |
| Zeaxanthin | β-Carotene | 0.178 | *** | |
| Zeaxanthin | Lycopene | 0.200 | *** | |
| β-Cryptoxanthin | α-Carotene | 0.549 | *** | |
| β-Cryptoxanthin | β-Carotene | 0.685 | *** | |
| β-Cryptoxanthin | Lycopene | 0.167 | *** | |
| α-Carotene | β-Carotene | 0.836 | *** | |
| α-Carotene | Lycopene | 0.346 | *** | |
| β-Carotene | Lycopene | 0.195 | *** | |

**Table S1.** Correlation of concentration of each carotenoid.

**p* < 0.05, ***p* < 0.01, ****p* < 0.001. Correlation analysis was performed using Spearman's rank-correlation coefficient.
